# Acidity and Antioxidant Activity of Cold Brew Coffee

**DOI:** 10.1038/s41598-018-34392-w

**Published:** 2018-10-30

**Authors:** Niny Z. Rao, Megan Fuller

**Affiliations:** Department of Chemistry and Biochemistry, Thomas Jefferson University, East Falls Campus, Philadelphia, PA 19144 USA

## Abstract

The acidity and antioxidant activity of cold brew coffee were investigated using light roast coffees from Brazil, two regions of Ethiopia, Columbia, Myanmar, and Mexico. The concentrations of three caffeoylquinic acid (CQA) isomers were also determined. Cold brew coffee chemistry was compared to that of hot brew coffee prepared with the same grind-to-coffee ratio. The pH values of the cold and hot brew samples were found to be comparable, ranging from 4.85 to 5.13. The hot brew coffees were found to have higher concentrations of total titratable acids, as well as higher antioxidant activity, than that of their cold brew counterparts. It was also noted that both the concentration of total titratable acids and antioxidant activity correlated poorly with total CQA concentration in hot brew coffee. This work suggests that the hot brew method tends to extract more non-deprotonated acids than the cold brew method. These acids may be responsible for the higher antioxidant activities observed in the hot brew coffee samples.

## Introduction

Cold brew coffee is a popular phenomenon that has recently invigorated the coffee industry, particularly in the warm summer months^1^. The domestic cold brew coffee market grew 580% from 2011 to 2016^2^. Roast Magazine reports a 460% increase in retail sales of refrigerated cold brew coffee in the United States from 2015 to 2017, generating $38 million in 2017 alone^3^. Cold brew coffee is made through a low-temperature, long-contact brewing method. Regional coffee vendors, such as Starbucks and Dunkin Donuts have marketed the product as tasting smoother and less bitter than traditional hot brewed coffees^4^. Consumer interest has also been spurred by a range of online health and lifestyle blogs publishing recipes and specific health claims for cold brew coffee. A recent article in *Healthy Living Made Simple*, a bimonthly publication with 4 million readers, states that “coffee brewed hot is far more acidic than cold-brewed, according to a number of scientific studies, and some say cold-brewed coffee even has a sweeter taste because of its lower acidity”^5^. A blog post on Coffee Brewing Methods makes several claims regarding the decreased acidity, decreased caffeine levels, and increased antioxidant content of cold brew coffee^6^. At the time of publication, there was very little published research on the chemistry of cold brew coffee and no published research on the health effects of cold brew coffee.

In fact, the health benefits and risks of traditional hot brew coffee consumption remain controversial. Coffee has long been associated with indigestion, heartburn, and other gastrointestinal symptoms. Epidemiological meta-analyses and patient-based experimentation have led to conflicting outcomes regarding the relationship between coffee consumption and gastrointestinal disorders. Early work by Thomas *et al*.^7^ found that coffee consumption in 20 healthy individuals and 16 patients with reflux esophagitis resulted in the decrease of lower esophageal sphincter (LES) pressure. The reduction of LES pressure, found in both cohorts following consumption of coffee with pH values of 4.5 and 7.0, could lead to aggravated heartburn symptoms^7^. Because the decrease in LES pressure occurred at both an acidic and neutral pH, acidity may not be the inciting factor in heartburn following coffee consumption. Two studies by Wendl *et al*.^8^ and Pehl *et al*.^9^ observed gastro-oesophageal reflux in asymptomatic individuals (n = 16) and patients with gastro-oesophageal disease (GERD) (n = 17), respectively, and found that both cohorts experienced decreased oesophageal reflux after consuming decaffeinated coffee, indicating that caffeine may responsible for coffee-related heartburn symptoms^8,9^. A recent population-based study of GERD patients (n = 317) and asymptomatic individuals (n = 182) found no association between GERD symptom frequency or severity and coffee consumption^10^. Kubo *et al*.’s work is in agreement with other meta-analyses that use patient-reported symptoms. Shimamato *et al*.^11^ used a large-scale multivariate analysis (n = 8,013) to evaluate coffee consumption as a contributor to the occurrence of gastric ulcers, duodenal ulcers, reflux esophagitis, and non-erosive reflux disease. Shimamato *et al*.^11^ found no significant relationships between coffee consumption and these four major acid-related gastrointestinal disorders^11^. Given the disagreement found in the literature regarding the health impacts of traditional hot coffee, it is understandable that the general public views coffee as a potential health risk despite significant evidence to the contrary.

Beyond gastrointestinal symptoms, coffee has been shown to correlate to multiple potential health benefits. A substantial umbrella review of numerous meta-analyses found no consistent evidence of harmful associations between coffee consumption and diverse health outcomes, with the exception of issues related to pregnancy and risk of bone fractures in women^12^. This work by Poole *et al*.^12^ evaluated previous research relating coffee consumption to cardiovascular health (including cardiovascular disease, coronary heart disease, and stroke), and found a reduction in health risks when three cups of coffee per day were consumed^13–16^. Poole *et al*.^12^ also found coffee consumption to be associated with decreased risk of liver^17,18^, metabolic^19,20^, and neurologic diseases^21,22^. The causal pathways for these chemoprotective associations between coffee consumption and disease are not well understood; however, recent studies of coffee have shown the beverage to exhibit high antioxidant capacity and anti-inflammatory effects. Work by Bakuradze *et al*.^23^ showed compounds present in coffee roast products - notably 5-caffeoylquinic acid, a type of chlorogenic acid, and caffeic acid - demonstrated direct antioxidant activity in HT-29 (human colon) cells^23^. The role of antioxidant compounds as radical-scavengers in the body is well-researched^24–26^, but the relationship between coffee consumption, antioxidant activity, and brewing methods is largely uncharacterized. A recent review by Naveed *et al*.^27^ further highlighted the therapeutic roles of chlorogenic acids in human health and called for further research in the area^27^. Work by Chu *et al*.^28^ found that roasted coffees contained higher antioxidant capacities and higher chlorogenic acid and phenolic concentrations than green coffee beans. Chu *et al*.'s work also found a strong correlation between neuroprotective efficacy of roasted coffee and total chlorogenic acid concentration^28^.

Despite the growing popularity of cold brew coffee, very little research has been published on its chemical attributes, including pH and total antioxidant activity, and associated health effects. An exhaustive literature search returned only four peer-reviewed studies related to cold brew coffee^29–32^. None of these studies provided enough information to either support or refute the health claims about cold brew coffee made by commercial coffee vendors and cold brew enthusiasts.

Given the significant growth of the cold brew coffee market and the potential importance of coffee’s bioactive compounds to human health, this study quantifies the pH, total titratable acidity, and total antioxidant capacity of cold brew coffee produced from grinds sourced from six different coffee-growing regions. Further, this research quantifies 5- caffeoylquinic (5-CQA), 4-caffeoylquinic (4-CQA), and 3-caffeoylquinic acid (3-CQA) in these cold brew coffees to better understand the relationship between CQA content and total antioxidant capacity of coffee. The total antioxidant capacity is a measure of radical scavenging capacity and was determined using a ABTS ((2,2′-Azino-bi(3-ethylbenzo-thiazonile-6-sulfonic acid) diammonium salt) radical cation decolourization assay. All coffees used in this study were light-to-medium roast, pre-ground beans purchased from a commercial vendor. Traditional hot brew coffees and cold brew coffees were compared to determine what, if any, differences exist in the acidity and antioxidant capacity of the resulting beverages as a function of brewing temperature and time.

## Results

### Hot Brew Coffee

The results from the hot brew coffee analyses are shown in Tables 1 and 2. The hot brew coffee samples analyzed in this study were found to have pH values ranging from 4.85 to 5.10. The Ethiopian-Ardi samples were observed to be the most acidic with a pH of 4.85 ± 0.09, whereas the Brazilian samples were the least acidic with a pH of 5.10 ± 0.02. Of the three CQA isomers analyzed, 5-CQA was found to have the highest concentration in all samples, in agreement with previous studies^33–39^. The Ethiopian-Ardi samples were also found to have the highest 5-CQA and total CQA concentration (1721 ± 99 mg/L of coffee and 3270 ± 90 mg/L of coffee, respectively). The Brazilian samples had the lowest 5-CQA and total CQA concentration (1261 ± 111 mg/L of coffee and 2503 ± 103 mg/L of coffee, respectively). The 3-CQA and 4-CQA concentrations were the highest in the Ethiopian-Ardi samples, while Myanmar samples contained the lowest concentration of these two isomers. Previous work by Moon *et al*.^35^ suggested that lower CQA concentration is correlated with a higher pH^35^. A similar trend was observed among the samples analyzed in this study, with a Pearson correlation coefficient of -0.70. These results agree well with pH data presented by Moon *et al*.^35^ for light roast hot brew coffees.

**Table 1:** Hot Brew Coffee Samples: concentration of 5-CQA, 4-CQA, 3-CQA, and total CQA concentration (milligrams per liter of brewed coffee) of hot brew coffee samples (Mean ± 95% Confidence Interval, n = 6).

	5-CQA (mg/L)	4-CQA (mg/L)	3-CQA (mg/L)	Total CQA (mg/L)
Brazilian	1261 ± 111	693 ± 45	550 ± 27	2503 ± 103
Ethiopian - Ardi	1721 ± 100	842 ± 22	707 ± 34	3270 ± 90
Ethiopian - Yirgz	1385 ± 285	635 ± 101	510 ± 78	2530 ± 261
Myanmar	1433 ± 341	595 ± 38	489 ± 30	2517 ± 277
Columbia	1429 ± 67	677 ± 22	562 ± 27	2669 ± 64
Mexico	1476 ± 111	721 ± 41	611 ± 38	2808 ± 105

**Table 2:** Hot Brew Coffee Samples: pH, total titratable acid concentration titrated to a pH of 6 and 8 (milliliters of 0.10 N NaOH per 40 milliliters of brewed coffee), and antioxidant activity (millimoles equivalence Trolox per liter of brewed coffee) of hot brew coffee samples (Mean ± 95% Confidence Interval, n = 6).

	pH	Total Acidity pH = 6 (mL of 0.10 N NaOH)	Total Acidity pH = 8 (mL of 0.10 N NaOH)	Antioxidant Activity (mmol equivalence Trolox/L coffee)
Brazilian	5.10 ± 0.02	3.17 ± 0.20	6.53 ± 0.38	18.34 ± 2.34
Ethiopian - Ardi	4.85 ± 0.09	3.62 ± 0.31	7.08 ± 0.74	19.95 ± 1.62
Ethiopian - Yirgz	4.96 ± 0.02	3.83 ± 0.33	7.45 ± 0.59	20.72 ± 3.12
Myanmar	4.92 ± 0.03	3.18 ± 0.75	6.40 ± 0.79	19.72 ± 1.17
Columbia	4.99 ± 0.10	4.27 ± 0.21	7.85 ± 0.06	19.98 ± 2.74
Mexico	4.95 ± 0.04	3.58 ± 0.41	6.68 ± 0.62	20.18 ± 1.65

The total titratable acidity (TA) of the coffees is expressed in mL of 0.10 N NaOH required to titrate 40 ml of coffee to a pH of 6 and a pH of 8. There have been multiple attempts to understand the chemical characteristics of coffee that cause the perception of bitterness in coffee. Bähre *et al*. has demonstrated that TA shows better correlation to sourness than pH^40^. Maier *et al*. found that the sourness of coffee correlates well with TA titrated to pH 6.0^41^. Balzer suggested that phenolic acids deprotonate at pH values greater than 8^42^. Thus, TA titrated to pH 8.0 may be better end point for titration^42^. Although sourness is not the focus of this study, TA titrated to these two endpoints may provide some insights about the acid contents in coffee. An earlier study by Gloess *et al*.^36^ found no correlation between pH and TA^36^. For hot brew coffee samples, Columbia coffee was found to have the highest concentration of total titratable acids at both pH of 6 and pH of 8. Brazilian and Myanmar samples were observed to have the lowest concentrations of total titratable acids at both pH of 6 and pH of 8. Data collected in this study showed little correlation between the pH and TA titrated to pH 6 (Pearson correlation coefficient = -0.15) and TA titrated to pH of 8 (Pearson correlation coefficient = -0.09) for hot brew coffee, in support of findings by Gloess *et al*.^36^.

Ethiopian-Yirgz samples were observed to have the highest antioxidant activity and Brazilian samples were observed to have the lowest antioxidant activity. In general, the results of this study for hot brew coffee agree well with the general body of knowledge regarding the chemical characterization of light-to-medium roast coffees, including CQA content^34,35^ and antioxidant activity^43–46^.

### Cold Brew Coffee

The results from the cold brew coffee analyses are shown in Tables 3 and 4. There is little published data to contextualize these results. However, comparison with the hot brew coffee characteristics in Table 1 point to the existence of chemical differences between cold and hot brew coffees prepared from the same coffee beans and extracted at the same ratio of water volume to grind weight. These data indicate that the temperature of the water used in brewing influences the release and diffusion of compounds in the resulting coffee beverage.

**Table 3:** Cold Brew Coffee Samples: concentration of 5-CQA, 4-CQA, 3-CQA, and total CQA concentration (milligrams per liter of brewed coffee) of cold brew coffee samples (Mean ± 95% Confidence Interval, n = 8).

	5-CQA (mg/L)	4-CQA (mg/L)	3-CQA (mg/L)	Total CQA (mg/L)
Brazilian	1124 ± 63	564 ± 23	513 ± 19	2201 ± 53
Ethiopian - Ardi	1133 ± 36	552 ± 14	464 ± 10	2149 ± 30
Ethiopian - Yirgz	1031 ± 127	480 ± 46	384 ± 34	1895 ± 104
Myanmar	912 ± 126	429 ± 28	355 ± 20	1697 ± 94
Columbia	1018 ± 157	488 ± 51	406 ± 41	1912 ± 127
Mexico	857 ± 138	416 ± 44	344 ± 35	1616 ± 111

**Table 4:** Cold Brew Coffee Samples: pH, total titratable acid concentration titrated to a pH of 6 and 8 (milliliters of 0.10 N NaOH per 40 milliliters of brewed coffee), and antioxidant activity (millimoles equivalence Trolox per liter of brewed coffee) of cold brew coffee samples (Mean ± 95% Confidence Interval, n = 6).

	pH	Total Acidity pH = 6 (mL of 0.10 N NaOH)	Total Acidity pH = 8 (mL of 0.10 N NaOH)	Antioxidant Activity (mmol equivalence Trolox/L coffee)
Brazilian	5.04 ± 0.16	2.83 ± 0.21	5.88 ± 0.31	16.10 ± 3.02
Ethiopian - Ardi	5.01 ± 0.02	2.55 ± 0.18	5.25 ± 0.19	17.45 ± 2.05
Ethiopian - Yirgz	4.96 ± 0.08	2.58 ± 0.18	5.18 ± 0.14	13.36 ± 0.99
Myanmar	5.13 ± 0.03	2.52 ± 0.14	5.32 ± 0.21	13.36 ± 2.85
Columbia	5.00 ± 0.05	2.93 ± 0.18	5.52 ± 0.32	15.33 ± 1.92
Mexico	5.08 ± 0.04	2.13 ± 0.11	4.75 ± 0.27	13.92 ± 2.69

The pH values of cold brew samples ranged from 4.96 to 5.13, with Ethiopian-Yirgz being the most acidic (pH = 4.96 ± 0.08) and Myanmar being the least acidic (5.13 ± 0.03). Similar to the hot brew counterparts, 5-CQA was found to be the most abundant CQA isomer in cold brew coffee. Brazilian samples were observed to have the highest concentration of all three CQA isomers whereas Mexican samples had the lowest CQA isomer concentrations. The correlation between pH and total CQA concentration in cold brew coffee is somewhat weak (Pearson correlation coefficient = -0.52).

In terms of total titratable acids, Mexican samples had the lowest concentration of total titratable acids at both pH of 6 and pH of 8. Columbia samples had the highest concentration of total titratable acids (TA) at pH of 6 and Brazilian samples had the highest concentration of total titratable acids at pH of 8. Similar to the hot brew samples, no correlation between pH and TA were observed for the cold brew samples. Ethiopian-Ardi samples were observed to have the highest antioxidant activity, Myanmar and Ethiopian-Yirgz samples had the lowest antioxidant activity. In general, the cold brew extracts were found to have pH values comparable to those of the hot brew extracts, but lower total acidity measures, lower total CQA concentrations, and lower total antioxidant activities.

### Hot and Cold Brew Comparisons

#### Total acidity and pH

Measurements of pH quantify the concentration of aqueous hydrogen ions at the time of analysis, providing a metric for the quantity of deprotonated acid molecules in a sample. Total titratable acidity (TA) is a measure of all acidic protons in a sample, including non-dissociated protons, that can be neutralized through the addition of a strong base.

**Figure 1:**
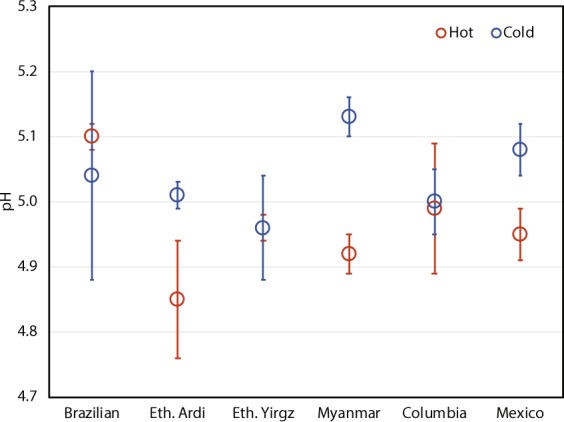
pH values of six coffee samples brewed using both hot and cold brewing methods. The error bars represent 95% confidence level.

Commercial vendors and coffee enthusiasts often suggest that cold brew and hot brew coffees boast different taste profiles due to differing acidity levels; and that cold brew coffee, being supposedly less acidic, may reduce gastrointestinal symptoms sometimes associated with coffee consumption^6,47–50^. This work found the pH measurements for all coffee samples tested to be comparable, ranging between 4.85 to 5.13. Varying the temperature of the extraction water did not result in distinguishable pH values between hot and cold brew coffees (Fig. 1).

**Figure 2:**
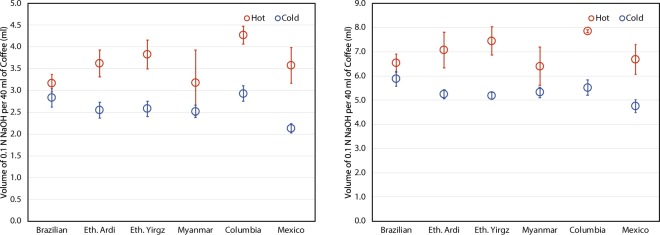
Total titratable acids of six coffee samples brewed using both hot and cold brewing methods measure at (*left*) pH of 6.0 and (*right*) pH of 8.0. The values are reported as milliliters of 0.1 NaOH per 40 milliliters of brewed coffee. The error bars represent 95% confidence level.

However, TA results indicate substantially different concentrations of total acidic compounds between hot and cold brew coffees. This research found hot coffee extracts to have larger measures of titratable acidity, indicating higher concentrations of extracted acids and/or additional acidic compounds not found in the cold brew coffee extracts (Fig. 2). The Pearson correlation coefficients for both hot and cold brew samples are less than 0.5. The lack of a correlation in this data agrees with the findings of Gloess *et al*.^36^ and suggests that pH is a poor measurement for the complex acid chemistry in both hot and cold brew coffee extracts.

**Figure 3:**
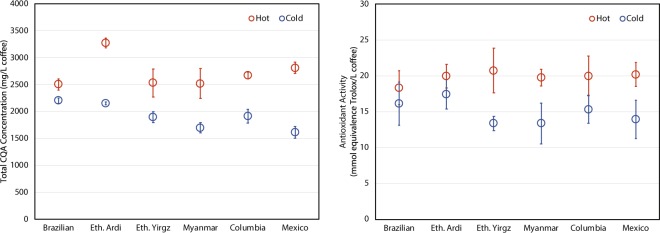
(*left*) 3-CGA concentration in milligrams per liter of brewed coffee and (*right*) antioxidant activity in mmol equivalent of Trolox per liter of brewed coffee of the six coffee samples brewed using both hot and cold brewing methods. The error bars represent 95% confidence level.

In general, these results suggest that cold and hot brew coffees are similar in their total concentrations of deprotonated acid compounds, but differ in the concentration and possibly the complexity of protonated acids at the pH of extraction. The total CQA concentration data, shown in Tables 1 and 3, found hot brew extracts to have higher total CQA concentrations (Fig. 3). This is one source of the difference in total titratable acidities (TA). The compounds present in hot brew coffee but absent from cold brew coffee may be larger molecules with temperature-dependent solubilities, and/or compounds with significant intermolecular forces that result in strong coffee matrix-compound attraction.

#### Antioxidant activity and Total CQA Concentration

The family of chlorogenic acid compounds are known to contribute significantly to the antioxidant activity of coffee. Work by Daglia *et al*.^51^ and Stadler *et al*.^52^ have found the polyphenolic compounds in coffee to have antioxidant and antiradical activity in radical-mediated mutagenic pathways. Given the importance of this family of compounds, correlations between antioxidant activity and CQA concentrations were analyzed.

**Figure 4:**
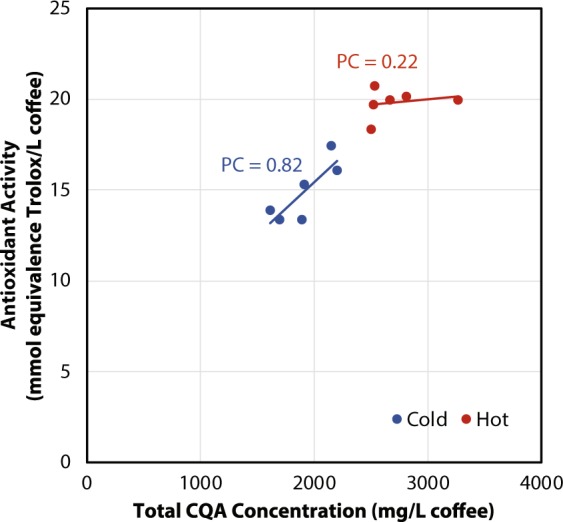
Relationship between 3-CGA concentration (mg/L brewed coffee) and antioxidant activity (mmol equivalent Trolox/L brewed coffee) for hot and cold brew coffees from the six regional coffee samples.

Similar to CQA data and TA, the data collected in this study indicated that hot brew extracts have higher antioxidant activity than their cold brew counterparts (Fig. 3). Figure 4 shows the relationship between antioxidant activity and total CQA concentration for hot and cold brew coffees. The cold brew samples were found to have a Pearson correlation coefficient of 0.82, indicating a relatively strong correlation between these two chemical characteristics. However, the antioxidant capacity and total CQA concentration of hot brew coffee were found to have a Pearson correlation coefficient of 0.22, indicating a much weaker relationship between antioxidant activity and chlorogenic acid concentration. Given that hot coffee extracts exhibited higher antioxidant activity than their cold brew counterparts, hot water must extract additional bioactive compounds. Hot brew coffees analyzed here were found to have increased concentrations of CQA isomers, and likely had increased concentrations of other chlorogenic acids. This may account for the difference in antioxidant activity between hot and cold brews, but there may be additional compounds responsible for this differential. The strong correlation between antioxidant activity and total CQA concentration in cold brew coffee suggests that CQA isomers are important drivers of cold brew coffee antioxidant activity.

## Discussion

Cold brew coffee extracts were found to have lower concentrations of acidic compounds and may be less chemically diverse than hot brew coffee extracts prepared from the same beans. This can be seen in both total acidity and antioxidant activity measurements. Hot coffee brews were found to have higher titratable acid levels, indicating higher concentrations of acidic compounds than in cold brew extracts, and/or additional acidic compounds not found in cold brew extracts. All cold brew coffee samples analyzed in this study were found to have lower titratable acid levels than their hot brew counterparts. Coffee is composed of dozens of low molecular mass compounds, including numerous carboxylic acids such as citric, malic, quinic, succinic, and gluconic acids^40,53^. While all of these acids are readily soluble in water, their ability to detach from the coffee matrix and diffuse through the intra- and intergranular pore spaces in room temperature water as is used in cold brew method is poorly understood.

Hot brew coffees had higher antioxidant capacities than their cold brew counterparts, indicating that additional radical-scavenging compounds and/or higher concentrations of such compounds were present in the hot brew samples. For cold brew coffee, a strong correlation was found between total CQA concentration and total antioxidant activity, while a weak correlation was seen for hot brew coffee. The total CQA concentration failed to correlate with antioxidant activity in hot brew coffee likely because those hot water extracts had a more diverse and complex chemistry than the cold brew samples. It can be assumed that many of the compounds absent from the cold brew coffees were acidic molecules, as the total acidity levels in the hot coffees were found to be greater.

This research finds that water temperature during aqueous extraction influences the transport of acidic molecules from the coffee matrix into the water phase substantially enough to alter the total titratable acidity and antioxidant activity of the resulting coffee beverage.

## Conclusions and Future Work

This research reveals important fundamental differences between hot and cold brew coffee that may have implications for possible health impacts on drinkers. It is often claimed by cold brew coffee enthusiasts that cold brew coffee has lower acidity than its hot brew counterparts, and thus may be a better alternative for those who suffer from gastrointestinal symptoms. This study suggests that the hot brew method tends to extract additional non-deprotonated acids in comparison to the cold brew method. These acids may be responsible for the higher antioxidant activities observed in hot brew coffee samples. Additionally, the chemical composition of hot brew coffee may be more diverse and complex than that of cold brew coffee. Additional research is needed to fully understand any possible differences in the health effects of coffee as a function of brewing temperature and time. The lower antioxidant capacity in cold brew coffees may decrease the chemoprotective benefits known to be associated with hot brew coffees.

To better understand the relationship between brewing temperature and chemical complexity of the resulting coffee, compound-specific analysis of the extracts is needed. There are several classes of compounds present in coffee extracts that may be the cause of the differences seen in hot and cold brew coffee in this study. One possible class of compounds that may influence pH and antioxidant activity levels are melanoidins. Melanoidin compounds are known to have antiradical properties and account for upwards of 25% of coffee’s dry matter^54,55^, however, they have not been characterized in cold brew coffees.

Previous studies have reported extensively on the chemical composition of coffee^34–37,42,56,57^. Future work to identify and quantify compounds present in hot and cold brew coffee would help to better elucidate the chemical differences between the two beverages. Further work could also be done to characterize the antioxidant activity of specific compounds and classes of compounds to better understand the role of brewing temperature on total antioxidant character of the resulting coffee beverages.

## Materials and Methods

### Materials

Pre-ground, light roast Brazilian, Colombian, Ethiopian, Mexican, and Myanmar coffees were purchased from commercial vendors. Coffee samples from two regions of Ethiopia (labeled as Ardi and Yirgz by the vendor) were analyzed separately.

5-Caffeoylquinic acid (5-CQA, CAS: 327-97-9), 4-CQA (CAS: 905-99-7), and 3-CQA (CAS: 906-33-2) were purchased from Sigma-Aldrich (Milwaukee, WI). HPLC grade methanol was obtained from Fisher Scientific (Nazareth, PA). Phosphoric acid (85% wt.) was obtained from Sigma-Aldrich (Milwaukee, WI) and diluted to 2.0 mM concentration using deionized (DI) water. Standard stock solutions of 2.5 mM Trolox (6-hydroxy-2,5,7,8-tetramethylchroman-2-carboxylic acid) were prepared in ethanol weekly. Trolox and ethanol were purchased from Sigma-Aldrich (Milwaukee, WI). ABTS˙^+^ (2,2′-azionbis(3-ethylbenzothiazoline-6-sulfonic acid) diammonium salt) radical cation solutions were prepared every 48 hours and stored in the dark at room temperature. The ABTS˙^+^ solution was allowed to stand for 12 hours after mixing to achieve maximal color formation. The potassium persulfate and ABTS reagents used to generate the radical solution were both obtained from Sigma-Aldrich (Milwaukee, WI). Standardized 0.1 N NaOH from Sigma-Aldrich (Milwaukee, WI) was used to find the total titratable acidity of each coffee. Filtered municipal tap water was used to brew the coffees. Analysis of this water, conducted by Penn State University’s Agricultural Analytical Services Laboratory, found the water to have a total hardness of 174 mg/L and a pH of 7.5.

### Methods

#### Cold brew experiments

The cold brewing process was carried out at room temperature (ranging from 21 °C to 25 °C over the experimental period) adapted from a home-brewing recipe published on *The New York Times*’*s* Cooking website^58^. A sample of 35.0 g of coffee was placed in 350 mL of carbon-filtered municipal water in a 32-ounce Mason jar fitted with a screw-top lid. The coffee was allowed to brew for 7 hours as suggested by our previous study^32^. The coffee samples were then filtered using the Hario V60 paper filter before analysis. Four samples were taken from each batch of filtered cold brew coffee, and each experiment was performed in duplicate (n = 8).

#### Hot brew experiments

Hot brew extraction was conducted using the same coffee-to-water ratio as was used in the cold brew method. The water was heated to boiling, then added to coffee grounds in a traditional French press carafe. The coffee samples were brewed for 6 minutes before filtering using the Hario V60 paper filter. It is noted that the samples at the time of filtering were different between hot and cold brew experiments. The experiments were designed to simulate typical brewing environments for consumption. Thus, the filtering process was not temperature controlled. Three samples were taken from each batch of filtered hot brew coffee, and each experiment was performed in duplicate (n = 6).

#### Sample Storage

Both cold brew and hot brew samples were freshly prepared for each experiment. All samples were analyzed within 10 minutes of brewing.

#### HPLC Analysis

Standard solutions and coffee extracts were analyzed using an adapted methodology reported in GL Sciences Technical Note No. 67^59^. An Agilent 1200 Series high-performance liquid chromatography system (HPLC) was fitted with a Supelco 5 µm column (15 cm × 4.6 cm) (Supelco, Bellefonte, PA) run at 40.0 °C with a mobile phase mixture of 75% mobile phase A and 25% mobile phase B (A: 95% 2.0 mM phosphoric acid and 5% methanol; B: 95% methanol and 5% 2.0 mM phosphoric acid). The flow rate was 1.0 mL/min with an injection volume of 10.0 µL. CQA isomers were detected using a diode array detector at 325 nm. 5-CQA was quantified via standard calibration curves. 4-CQA and 3-CQA standards were used to determine the retention time of each isomer. Quantitation of the other CQA isomers was accomplished using the area of 5-CQA standard combined with the respective molar extinction coefficients of other two isomers as reported previously^33,34,38^.

#### Total acidity and pH measurements

The pH of each brewed coffee sample was measured with a Mettler Toledo FiveEasy^TM^ F20 benchtop pH/mV meter. A 40 mL aliquot of coffee brew was titrated with 0.1 N NaOH at 22 °C to a pH of 6.0 and a pH of 8.0.

#### Antioxidant activity measurements

Total antioxidant activity of hot and cold brew coffees was determined using an ABTS radical cation decolorization assay modified from Re *et al*. and Vignoli *et al*.^60,61^. To summarize the procedure, a stock solution of ABTS˙^+^ was made by mixing equal parts 7.0 mM ABTS and 2.45 mM potassium persulfate to form the ABTS˙^+^ radical cation. The mixture was allowed to stand in the dark at room temperature for 14 to 16 hours to reach optimal absorbance at 734 nm. A dilute working solution of ABTS˙^+^ with an absorbance between 0.80 and 0.90 at 734 nm was made by diluting the stock solution with DI water. Trolox standards were tested by mixing 30 µL of 2.5 mM Trolox solution with 4.0 mL of diluted ABTS˙^+^ solution and allowing to stand for 6 minutes. The resulting solution was analyzed by UV-Vis spectroscopy at 734 nm using a Thermo Scientific Evolution 201 spectrophotometer, and ABTS˙^+^ scavenging capacity was determined by absorbance difference between the working standard and the Trolox - ABTS˙^+^ sample.

Filtered coffee samples were diluted 1:2 with DI water and centrifuged at 8000 rev/min for 2 minutes to further remove any particulates from the sample. A 5.0 µL aliquot of coffee was pipetted into 4.0 mL of the dilute ABTS˙^+^ and allowed to stand for 6 minutes. The resulting solution was analyzed by UV-Vis following the procedure for the Trolox standards. The total antioxidant capacity of each coffee sample was calculated as mmol Trolox equivalent per liter of brewed coffee.

#### Statistical analysis

ANOVA (Table S1) and two-tailed student’s t-test (Table S2) were employed to determine similarities in antioxidant activities, pH values, total acidities, and equilibrium concentrations of CQA with consideration to the origin of the coffee and brewing method. The output of the statistical analysis is included in the supplementary information.

## Electronic supplementary material

Supporting Information

## Data Availability

All data generated or analyzed during this study are included in this published article (and its Supplementary Information files).

## References

[1] Berry, D. Competition heats up in the cold brew category. *Food Business New*s, Available at: https://www.foodbusinessnews.net/articles/7365-competition-heats-up-in-the-cold-brew-category (Accessed: 14th May 2018) (2016).

[2] Sisel, E. The Strength of Cold Brew. *Mintel*, Available at: http://www.mintel.com/blog/drink-market-news/the-strength-of-cold-brew (Accessed: 14th May 2018) (2016).

[3] Brown, N. New US Coffee Shop Growth Slows as RTD and Cold Brew Accelerate, According to Mintel. *Roast Magazin*e, Available at: https://dailycoffeenews.com/2017/10/03/new-us-coffee-shop-growth-slows-as-rtd-and-cold-brew-accelerate-according-to-mintel/ (Accessed: 14th May 2018) (2017).

[4] Meyer, D. Dunkin’ Donuts Is Giving Away Free Cold Brew Coffee Today. Here’s How To Get Yours. *Fortune*, Available at: http://fortune.com/2018/04/06/dunkin-donuts-free-coffee-cold-brew/ (Accessed: 14th May 2018) (2018).

[5] Miesse, M. Some benefits of drinking cold-brew coffee. *Healthy Living Made Simple*, Available at: http://healthylivingmadesimple.com/benefits-drinking-cold-brew-coffee/#respond (Accessed: 14th May 2018).

[6] Bodnariuc, D. 5 Health Benefits Of Cold Brew Coffee – Why Is Cold Brew Better Than Drip Coffee. *Coffee Brewing Method*s, Available at: https://coffee-brewing-methods.com/cold-brew/health-benefits-of-cold-brew-coffee/ (Accessed: 14th May 2018) (2017).

[7] Thomas FB, Steinbaugh JT, Fromkes JJ, Mekhjian HS, Caldwell JH (1980). Inhibitory effect of coffee on lower esophageal sphincter pressure. Gastroenterology.

[8] Wendl B, Pfeiffer A, Pehl C, Schmidt T, Kaess H (1994). Effect of decaffeination of coffee or tea on gastro-oesophageal reflux. Aliment. Pharmacol. Ther..

[9] Pehl C, Pfeiffer A, Wendl B, Kaess H (1997). The effect of decaffeination of coffee on gastro-oesophageal reflux in patients with reflux disease. Aliment. Pharmacol. Ther..

[10] Kubo A, Block G, Quesenberry CP, Buffler P, Corley DA (2014). Dietary guideline adherence for gastroesophageal reflux disease. BMC Gastroenterol..

[11] Shimamoto T (2013). No Association of Coffee Consumption with Gastric Ulcer, Duodenal Ulcer, Reflux Esophagitis, and Non-Erosive Reflux Disease: A Cross-Sectional Study of 8,013 Healthy Subjects in Japan. PLoS One.

[12] Poole R (2017). Coffee consumption and health: umbrella review of meta-analyses of multiple health outcomes. BMJ.

[13] Ding M, Bhupathiraju SN, Satija A, van Dam RM, Hu FB (2014). Long-term coffee consumption and risk of cardiovascular disease: a systematic review and a dose-response meta-analysis of prospective cohort studies. Circulation.

[14] Grosso G (2016). Coffee consumption and risk of all-cause, cardiovascular, and cancer mortality in smokers and non-smokers: a dose-response meta-analysis. Eur. J. Epidemiol..

[15] Larsson, S. C., Drca, N., Jensen-Urstad, M. & Wolk, A. Coffee consumption is not associated with increased risk of atrial fibrillation: results from two prospective cohorts and a meta-analysis. *BMC Med*. **13** (2015). 10.1186/s12916-015-0447-8 PMC4579587 26394673

[16] Lippi G, Mattiuzzi C, Franchini M (2015). Venous thromboembolism and coffee: critical review and meta-analysis. Ann Transl Med.

[17] Liu F (2015). Coffee Consumption Decreases Risks for Hepatic Fibrosis and Cirrhosis: A Meta-Analysis. PLoS One.

[18] Wijarnpreecha K, Thongprayoon C, Ungprasert P (2017). Coffee consumption and risk of nonalcoholic fatty liver disease. Eur. J. Gastroenterol. Hepatol..

[19] Ding M, Bhupathiraju SN, Chen M, van Dam RM, Hu FB (2014). Caffeinated and decaffeinated coffee consumption and risk of type 2 diabetes: a systematic review and a dose-response meta-analysis. Diabetes Care.

[20] Jiang X, Zhang D, Jiang W (2014). Coffee and caffeine intake and incidence of type 2 diabetes mellitus: a meta-analysis of prospective studies. Eur. J. Nutr..

[21] Hernán MA, Takkouche B, Caamaño-Isorna F, Gestal-Otero JJ (2002). A meta-analysis of coffee drinking, cigarette smoking, and the risk of Parkinson’s disease. Ann. Neurol..

[22] Wang L, Shen X, Wu Y, Zhang D (2016). Coffee and caffeine consumption and depression: A meta-analysis of observational studies. Aust. N. Z. J. Psychiatry.

[23] Bakuradze T (2010). Antioxidant effectiveness of coffee extracts and selected constituents in cell-free systems and human colon cell lines. Mol. Nutr. Food Res..

[24] Barber DA, Harris SR (1994). Oxygen free radicals and antioxidants: a review. Am. Pharm..

[25] Choudhari SK, Chaudhary M, Gadbail AR, Sharma A, Tekade S (2014). Oxidative and antioxidative mechanisms in oral cancer and precancer: a review. Oral Oncol..

[26] Valko M (2007). Free radicals and antioxidants in normal physiological functions and human disease. Int. J. Biochem. Cell Biol..

[27] Naveed M (2018). Chlorogenic acid (CGA): A pharmacological review and call for further research. Biomed. Pharmacother..

[28] Chu Y-F (2009). Roasted coffees high in lipophilic antioxidants and chlorogenic acid lactones are more neuroprotective than green coffees. J. Agric. Food Chem..

[29] Kim AR, Kim JS (2014). Flavor Contributing Nonvolatile Chemical and Sensory Characterization of Cold Water Extraction-based Coffee by Different ExtractionMethods (Dripping vs Steeping) and Time. Journal of The Korea Society for Coffee Industry.

[30] Lane S, Palmer J, Christie B, Ehlting J, Le C (2017). Can Cold Brew Coffee Be Convenient? A Pilot Study For Caffeine Content in Cold Brew Coffee Concentrate Using High Performance Liquid Chromatography. The Arbutus Review.

[31] Shin K-S (2017). The Chemical Characteristics and Immune-Modulating Activity of Polysaccharides Isolated from Cold-Brew Coffee. Prev Nutr Food Sci.

[32] Fuller M, Rao NZ (2017). The Effect of Time, Roasting Temperature, and Grind Size on Caffeine and Chlorogenic Acid Concentrations in Cold Brew Coffee. Sci. Rep..

[33] Trugo LC, Macrae R (1984). Chlorogenic acid composition of instant coffees. Analyst.

[34] Farah A, de Paulis T, Trugo LC, Martin PR (2005). Effect of roasting on the formation of chlorogenic acid lactones in coffee. J. Agric. Food Chem..

[35] Moon J-K, Yoo HS, Shibamoto T (2009). Role of roasting conditions in the level of chlorogenic acid content in coffee beans: correlation with coffee acidity. J. Agric. Food Chem..

[36] Gloess AN (2013). Comparison of nine common coffee extraction methods: instrumental and sensory analysis. Eur. Food Res. Technol..

[37] Fujioka K, Shibamoto T (2008). Chlorogenic acid and caffeine contents in various commercial brewed coffees. Food Chem..

[38] Monente C, Ludwig IA, Irigoyen A, De Peña M-P, Cid C (2015). Assessment of total (free and bound) phenolic compounds in spent coffee extracts. J. Agric. Food Chem..

[39] Ludwig IA (2012). Extraction of coffee antioxidants: Impact of brewing time and method. Food Res. Int..

[40] Bähre F, Maier HG (1996). Electrophoretic clean-up of organic acids from coffee for the GC/MS analysis. Fresenius J. Anal. Chem..

[41] Maier HG, Balcke C, Thies F-C (1983). Die Säuren Des Kaffees. VI. Abhängigkeit des sauren Geschmacks von pH-Vert und Säuregrad. Lebensm. Gerichtl. Chem..

[42] Balzer, H. H. Acids in coffee. In *Coffee*: *Recent Development* (eds Clarke, R. J. & Vitzthum, O. G.) **1**, 18–32 (Oxford, England: Blackwell Science, 2001).

[43] del Castillo MD, Ames JM, Gordon MH (2002). Effect of roasting on the antioxidant activity of coffee brews. J. Agric. Food Chem..

[44] Cämmerer B, Kroh LW (2006). Antioxidant activity of coffee brews. Eur. Food Res. Technol..

[45] Vignoli JA, Bassoli DG, Benassi MT (2011). Antioxidant activity, polyphenols, caffeine and melanoidins in soluble coffee: The influence of processing conditions and raw material. Food Chem..

[46] Richelle M, Tavazzi I, Offord E (2001). Comparison of the antioxidant activity of commonly consumed polyphenolic beverages (coffee, cocoa, and tea) prepared per cup serving. J. Agric. Food Chem..

[47] How Coffee Lovers Manage Acid Reflux | Café Altura. *Cafe Altura*, Available at: https://cafealtura.com/coffee-and-acid-reflux/ (Accessed: 31st May 2018) (2014).

[48] GERD and Caffeine: Are Coffee and Tea Off Limits? *Healthline*, Available at: https://www.healthline.com/health/gerd/coffee-tea (Accessed: 31st May 2018) (2015).

[49] Cold Brew Coffee Lowers HeartburnRisk - How to Treat Heartburn. *How to Treat Heartburn*, Available at: https://howtotreatheartburn.com/cold-brew-coffee-lowers-heartburn-chances/ (Accessed: 31st May 2018) (2015).

[50] Rodrigo. Can Coffee Cause Acid Reflux? Yes! 7 Ways to Avoid It. *LittleCoffee Plac*e, Available at: https://www.littlecoffeeplace.com/coffee-acid-reflux (Accessed: 31st May 2018) (2018).

[51] Daglia M (2004). *In vitro* and *ex vivo* antihydroxyl radical activity of green and roasted coffee. J. Agric. Food Chem..

[52] Stadler RH, Turesky RJ, Müller O, Markovic J, Leong-Morgenthaler PM (1994). The inhibitory effects of coffee on radical-mediated oxidation and mutagenicity. Mutat. Res..

[53] Bähre, F. & Maier, H. G. New non-volatile acids in coffee. *Deutsche Lebensmittel-Rundschau* (*Germany*) **95** (1999).

[54] Borrelli RC, Visconti A, Mennella C, Anese M, Fogliano V (2002). Chemical characterization and antioxidant properties of coffee melanoidins. J. Agric. Food Chem..

[55] Hofmann T, Bors W, Stettmaier K (1999). Radical-assisted melanoidin formation during thermal processing of foods as well as under physiological conditions. J. Agric. Food Chem..

[56] Niseteo T, Komes D, Belščak-Cvitanović A, Horžić D, Budeč M (2012). Bioactive composition and antioxidant potential of different commonly consumed coffee brews affected by their preparation technique and milk addition. Food Chem..

[57] Moon J-K, Shibamoto T (2009). Role of roasting conditions in the profile of volatile flavor chemicals formed from coffee beans. J. Agric. Food Chem..

[58] Times, T. N. Y. Cold-Brewed Iced Coffee Recipe. *The New York Times*.

[59] Tanaka, Y. *Analysis of chlorogenic acid in coffee by HPLC*. (GL Science Inc).

[60] Re R (1999). Antioxidant activity applying an improved ABTS radical cation decolorization assay. Free Radic. Biol. Med..

[61] Vignoli JA, Viegas MC, Bassoli DG, de Toledo Benassi M (2014). Roasting process affects differently the bioactive compounds and the antioxidant activity of arabica and robusta coffees. Food Res. Int..

