# Supplementary material for: Acidity and Antioxidant Activity of Cold Brew Coffee
Source: Sci Rep. 2018 Oct 30;8:16030. doi: 10.1038/s41598-018-34392-w (PMC6207714; doi:10.1038/s41598-018-34392-w)
Supplement: Supplementary file 1 — Supporting Information [file 41598_2018_34392_MOESM1_ESM.docx]

**Supporting Information:**

**Acidity and Antioxidant Activity of Cold Brew Coffee**

Niny Z. Rao^1,*^, Megan Fuller^1^

^1^Department of Chemistry and Biochemistry, Thomas Jefferson University, East Falls Campus, Philadelphia, PA 19144, USA

* Corresponding Author

Niny Z. Rao, Ph.D.

Thomas Jefferson University

East Falls Campus

Philadelphia, PA 19144

Telephone: (215) 951-0906

E-mail: niny.rao@jefferson.edu

**Figure S1**. Sample chromatogram of CGQ isomers at 325 nm (top) in reference standard solution and (*bottom*) in a coffee extract. The detector response in absorption units (mAU) is plotted against the elution time in minutes.

**
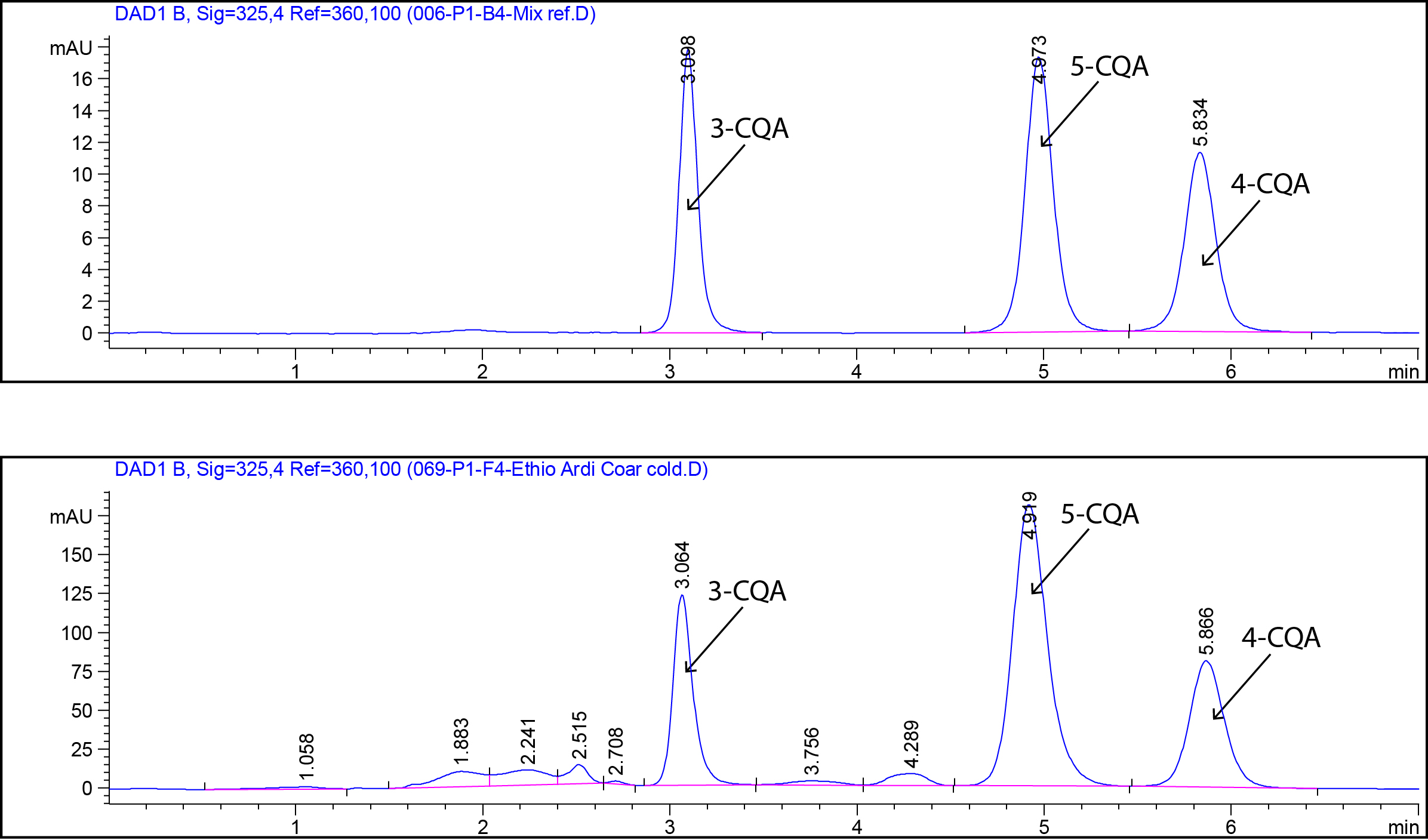
**

**Table S1a**. One way ANOVA for the comparison of the pH values of six coffee samples using the cold brew method.

| ***Analysis of Variance (One-Way)*** | | |  |  |  |  |
| --- | --- | --- | --- | --- | --- | --- |
|  |  |  |  |  |  |  |
| **SUMMARY** |  |  |  |  |  |  |
| *Groups* | *Count* | *Sum* | *Average* | *Variance* |  |  |
| Brazil | 6 | 30.21 | 5.035 | 0.03899 |  |  |
| Ethiopian Ardi | 6 | 29.78 | 4.9633333 | 0.0102267 |  |  |
| Ethiopian Yirgz | 6 | 30.07 | 5.0116667 | 0.0007767 |  |  |
| Myanmar | 6 | 30.79 | 5.1316667 | 0.0019767 |  |  |
| Columbia | 6 | 29.98 | 4.9966667 | 0.0040267 |  |  |
| Mexico | 6 | 30.45 | 5.075 | 0.00215 |  |  |
| **ANOVA** |  |  |  |  |  |  |
| *Source of Variation* | *SS* | *df* | *MS* | *F* | *P-value* | *F crit* |
| Between Groups | 0.1085556 | 5 | 0.0217111 | 2.2403119 | 0.0760067 | 2.5335545 |
| Within Groups | 0.2907333 | 30 | 0.0096911 |  |  |  |
|  |  |  |  |  |  |  |
| Total | 0.3992889 | 35 |  |  |  |  |

**Table S1b**. One way ANOVA for the comparison of the pH values of the six coffee samples using the hot brew method.

| ***Analysis of Variance (One-Way)*** | | |  |  |  |  |
| --- | --- | --- | --- | --- | --- | --- |
|  |  |  |  |  |  |  |
| **SUMMARY** |  |  |  |  |  |  |
| *Groups* | *Count* | *Sum* | *Average* | *Variance* |  |  |
| Brazil | 6 | 30.61 | 5.1016667 | 0.0002567 |  |  |
| Ethiopian Ardi | 6 | 29.51 | 4.9183333 | 0.0009367 |  |  |
| Ethiopian Yirgz | 6 | 29.76 | 4.96 | 0.01216 |  |  |
| Myanmar | 6 | 29.68 | 4.9466667 | 0.0012667 |  |  |
| Columbia | 6 | 29.09 | 4.8483333 | 0.0153767 |  |  |
| Mexico | 6 | 29.95 | 4.9916667 | 0.0020967 |  |  |
| **ANOVA** |  |  |  |  |  |  |
| *Source of Variation* | *SS* | *df* | *MS* | *F* | *P-value* | *F crit* |
| Between Groups | 0.2126889 | 5 | 0.0425378 | 7.9526381 | 7.228E-05 | 2.5335545 |
| Within Groups | 0.1604667 | 30 | 0.0053489 |  |  |  |
|  |  |  |  |  |  |  |
| Total | 0.3731556 | 35 |  |  |  |  |

**Table S2a**. One way ANOVA for the comparison of the total acidity of the six coffee samples titrated to pH of 6.0 using the cold brew method.

| ***Analysis of Variance (One-Way)*** | | |  |  |  |  |
| --- | --- | --- | --- | --- | --- | --- |
|  |  |  |  |  |  |  |
| **SUMMARY** |  |  |  |  |  |  |
| *Groups* | *Count* | *Sum* | *Average* | *Variance* |  |  |
| Brazil | 6 | 17 | 2.8333333 | 0.0666667 |  |  |
| Ethiopian Ardi | 6 | 15.5 | 2.5833333 | 0.0496667 |  |  |
| Ethiopian Yirgz | 6 | 15.3 | 2.55 | 0.047 |  |  |
| Myanmar | 6 | 15.1 | 2.5166667 | 0.0296667 |  |  |
| Columbia | 6 | 17.6 | 2.9333333 | 0.0546667 |  |  |
| Mexico | 6 | 12.8 | 2.1333333 | 0.0186667 |  |  |
| **ANOVA** |  |  |  |  |  |  |
| *Source of Variation* | *SS* | *df* | *MS* | *F* | *P-value* | *F crit* |
| Between Groups | 2.3558333 | 5 | 0.4711667 | 10.6145181 | 6.143E-06 | 2.5335545 |
| Within Groups | 1.3316667 | 30 | 0.0443889 |  |  |  |
|  |  |  |  |  |  |  |
| Total | 3.6875 | 35 |  |  |  |  |

**Table S2b**. One way ANOVA for the comparison of the total acidity of the six coffee samples titrated to pH of 6.0 using the hot brew method.

| ***Analysis of Variance (One-Way)*** | | |  |  |  |  |
| --- | --- | --- | --- | --- | --- | --- |
|  |  |  |  |  |  |  |
| **SUMMARY** |  |  |  |  |  |  |
| *Groups* | *Count* | *Sum* | *Average* | *Variance* |  |  |
| Brazil | 6 | 19 | 3.1666667 | 0.0626667 |  |  |
| Ethiopian Ardi | 6 | 23 | 3.8333333 | 0.1666667 |  |  |
| Ethiopian Yirgz | 6 | 21.7 | 3.6166667 | 0.1536667 |  |  |
| Myanmar | 6 | 19.1 | 3.1833333 | 0.8776667 |  |  |
| Columbia | 6 | 25.6 | 4.2666667 | 0.0666667 |  |  |
| Mexico | 6 | 21.5 | 3.5833333 | 0.2576667 |  |  |
| **ANOVA** |  |  |  |  |  |  |
| *Source of Variation* | *SS* | *df* | *MS* | *F* | *P-value* | *F crit* |
| Between Groups | 5.1625 | 5 | 1.0325 | 3.908517 | 0.007576 | 2.533555 |
| Within Groups | 7.925 | 30 | 0.2641667 |  |  |  |
|  |  |  |  |  |  |  |
| Total | 13.0875 | 35 |  |  |  |  |

**Table S3a**. One way ANOVA for the comparison of the total acidity of the six coffee samples titrated to pH of 8.0 using the cold brew method.

| ***Analysis of Variance (One-Way)*** | | |  |  |  |  |
| --- | --- | --- | --- | --- | --- | --- |
|  |  |  |  |  |  |  |
| **SUMMARY** |  |  |  |  |  |  |
| *Groups* | *Count* | *Sum* | *Average* | *Variance* |  |  |
| Brazil | 6 | 35.3 | 5.8833333 | 0.1496667 |  |  |
| Ethiopian Ardi | 6 | 31.1 | 5.1833333 | 0.0296667 |  |  |
| Ethiopian Yirgz | 6 | 31.5 | 5.25 | 0.059 |  |  |
| Myanmar | 6 | 31.9 | 5.3166667 | 0.0696667 |  |  |
| Columbia | 6 | 33.1 | 5.5166667 | 0.1616667 |  |  |
| Mexico | 6 | 28.5 | 4.75 | 0.115 |  |  |
| **ANOVA** |  |  |  |  |  |  |
| *Source of Variation* | *SS* | *df* | *MS* | *F* | *P-value* | *F crit* |
| Between Groups | 4.2266667 | 5 | 0.8453333 | 8.6750285 | 3.56E-05 | 2.5335545 |
| Within Groups | 2.9233333 | 30 | 0.0974444 |  |  |  |
|  |  |  |  |  |  |  |
| Total | 7.15 | 35 |  |  |  |  |

**Table S3b**. One way ANOVA for the comparison of the total acidity of the six coffee samples titrated to pH of 8.0 using the hot brew method.

| ***Analysis of Variance (One-Way)*** | | |  |  |  |  |
| --- | --- | --- | --- | --- | --- | --- |
|  |  |  |  |  |  |  |
| **SUMMARY** |  |  |  |  |  |  |
| *Groups* | *Count* | *Sum* | *Average* | *Variance* |  |  |
| Brazil | 6 | 39.2 | 6.5333333 | 0.2186667 |  |  |
| Ethiopian Ardi | 6 | 44.7 | 7.45 | 0.551 |  |  |
| Ethiopian Yirgz | 6 | 42.5 | 7.0833333 | 0.8376667 |  |  |
| Myanmar | 6 | 38.4 | 6.4 | 0.98 |  |  |
| Columbia | 6 | 47.1 | 7.85 | 0.007 |  |  |
| Mexico | 6 | 40.1 | 6.6833333 | 0.6016667 |  |  |
| **ANOVA** |  |  |  |  |  |  |
| *Source of Variation* | *SS* | *df* | *MS* | *F* | *P-value* | *F crit* |
| Between Groups | 9.66 | 5 | 1.932 | 3.6270338 | 0.0110106 | 2.5335545 |
| Within Groups | 15.98 | 30 | 0.5326667 |  |  |  |
|  |  |  |  |  |  |  |
| Total | 25.64 | 35 |  |  |  |  |

**Table S4a**. One way ANOVA for the comparison of the antioxidant activities of the six coffee samples using the cold brew method.

| ***Analysis of Variance (One-Way)*** | | |  |  |  |  |
| --- | --- | --- | --- | --- | --- | --- |
|  |  |  |  |  |  |  |
| **SUMMARY** |  |  |  |  |  |  |
| *Groups* | *Count* | *Sum* | *Average* | *Variance* |  |  |
| Brazil | 6 | 96.65693 | 16.10949 | 9.142544 |  |  |
| Ardi | 6 | 104.6715 | 17.44526 | 4.203357 |  |  |
| Yirgz | 6 | 80.18978 | 13.36496 | 0.974692 |  |  |
| Myanmar | 6 | 80.18978 | 13.36496 | 8.113698 |  |  |
| Columbia | 6 | 91.9708 | 15.32847 | 3.693409 |  |  |
| Mexico | 6 | 83.51825 | 13.91971 | 7.222953 |  |  |
| **ANOVA** |  |  |  |  |  |  |
| *Source of Variation* | *SS* | *df* | *MS* | *F* | *P-value* | *F crit* |
| Between Groups | 82.77287 | 5 | 16.55457 | 2.978276 | 0.026733 | 2.533555 |
| Within Groups | 166.7533 | 30 | 5.558442 |  |  |  |
|  |  |  |  |  |  |  |
| Total | 249.5261 | 35 |  |  |  |  |

**Table S4b**. One way ANOVA for the comparison of the antioxidant activities of the six coffee samples using the hot brew method.

| ***Analysis of Variance (One-Way)*** | | |  |  |  |  |
| --- | --- | --- | --- | --- | --- | --- |
|  |  |  |  |  |  |  |
| **SUMMARY** |  |  |  |  |  |  |
| *Groups* | *Count* | *Sum* | *Average* | *Variance* |  |  |
| Brazil | 6 | 110.0365 | 18.33942 | 5.464686 |  |  |
| Ardi | 6 | 119.6934 | 19.94891 | 2.618339 |  |  |
| Yirgz | 6 | 124.292 | 20.71533 | 9.756769 |  |  |
| Myanmar | 6 | 119.5182 | 19.91971 | 1.378251 |  |  |
| Columbia | 6 | 119.781 | 19.9635 | 7.516799 |  |  |
| Mexico | 6 | 121.0949 | 20.18248 | 2.730098 |  |  |
| **ANOVA** |  |  |  |  |  |  |
| *Source of Variation* | *SS* | *df* | *MS* | *F* | *P-value* | *F crit* |
| Between Groups | 19.01142 | 5 | 3.802283 | 0.774266 | 0.575907 | 2.533555 |
| Within Groups | 147.3247 | 30 | 4.910824 |  |  |  |
|  |  |  |  |  |  |  |
| Total | 166.3361 | 35 |  |  |  |  |

**Table S5a**. One way ANOVA for the comparison of the 5-CQA concentration of the six coffee samples using the cold brew method.

| ***Analysis of Variance (One-Way)*** | | |  |  |  |  |
| --- | --- | --- | --- | --- | --- | --- |
|  |  |  |  |  |  |  |
| **SUMMARY** |  |  |  |  |  |  |
| *Groups* | *Count* | *Sum* | *Average* | *Variance* |  |  |
| Brazil | 8 | 8993.2789 | 1124.1599 | 3907.8429 |  |  |
| Ethiopian Ardi | 8 | 9067.6908 | 1133.4614 | 1320.3512 |  |  |
| Ethiopian Yirgz | 8 | 8244.3591 | 1030.5449 | 15916.908 |  |  |
| Myanmar | 8 | 7297.6476 | 912.20595 | 15956.78 |  |  |
| Columbia | 8 | 8141.1426 | 1017.6428 | 24546.228 |  |  |
| Mexico | 8 | 6853.0965 | 856.63706 | 19065.706 |  |  |
| **ANOVA** |  |  |  |  |  |  |
| *Source of Variation* | *SS* | *df* | *MS* | *F* | *P-value* | *F crit* |
| Between Groups | 494430.2 | 5 | 98886.04 | 7.350863 | 5.02E-05 | 2.437693 |
| Within Groups | 564996.7 | 42 | 13452.3 |  |  |  |
|  |  |  |  |  |  |  |
| Total | 1059427 | 47 |  |  |  |  |

**Table S5b**. One way ANOVA for the comparison of the 5-CQA concentration of the six coffee samples using the hot brew method.

| ***Analysis of Variance (One-Way)*** | | |  |  |  |  |
| --- | --- | --- | --- | --- | --- | --- |
|  |  |  |  |  |  |  |
| **SUMMARY** |  |  |  |  |  |  |
| *Groups* | *Count* | *Sum* | *Average* | *Variance* |  |  |
| Brazil | 6 | 7565.53 | 1260.922 | 12235.09 |  |  |
| Ethiopian Ardi | 6 | 10323.57 | 1720.595 | 9928.717 |  |  |
| Ethiopian Yirgz | 6 | 8312.242 | 1385.374 | 81187.67 |  |  |
| Myanmar | 6 | 8599.136 | 1433.189 | 116067.4 |  |  |
| Columbia | 6 | 8576.572 | 1429.429 | 4431.711 |  |  |
| Mexico | 6 | 8854.057 | 1475.676 | 12425.34 |  |  |
| **ANOVA** |  |  |  |  |  |  |
| *Source of Variation* | *SS* | *df* | *MS* | *F* | *P-value* | *F crit* |
| Between Groups | 687057.1 | 5 | 137411.4 | 3.489431 | 0.013252 | 2.533555 |
| Within Groups | 1181379 | 30 | 39379.32 |  |  |  |
|  |  |  |  |  |  |  |
| Total | 1868437 | 35 |  |  |  |  |

**Table S6a**. One way ANOVA for the comparison of the 4-CQA concentration of the six coffee samples using the cold brew method.

| ***Analysis of Variance (One-Way)*** | | |  |  |  |  |
| --- | --- | --- | --- | --- | --- | --- |
|  |  |  |  |  |  |  |
| **SUMMARY** |  |  |  |  |  |  |
| *Groups* | *Count* | *Sum* | *Average* | *Variance* |  |  |
| Brazil | 8 | 4510.162 | 563.770 | 1114.946 |  |  |
| Ethiopian Ardi | 8 | 4412.906 | 551.613 | 418.631 |  |  |
| Ethiopian Yirgz | 8 | 3843.415 | 480.427 | 4324.674 |  |  |
| Myanmar | 8 | 3430.989 | 428.874 | 1672.066 |  |  |
| Columbia | 8 | 3901.144 | 487.643 | 5442.880 |  |  |
| Mexico | 8 | 3324.372 | 415.546 | 3962.860 |  |  |
| **ANOVA** |  |  |  |  |  |  |
| *Source of Variation* | *SS* | *df* | *MS* | *F* | *P-value* | *F crit* |
| Between Groups | 148725.50 | 5 | 29745.10 | 10.54 | 1.33E-06 | 2.43769 |
| Within Groups | 118552.39 | 42 | 2822.68 |  |  |  |
|  |  |  |  |  |  |  |
| Total | 267277.90 | 47 |  |  |  |  |

**Table S6b**. One way ANOVA for the comparison of the 4-CQA concentration of the six coffee samples using the hot brew method.

| ***Analysis of Variance (One-Way)*** | | |  |  |  |  |
| --- | --- | --- | --- | --- | --- | --- |
|  |  |  |  |  |  |  |
| **SUMMARY** |  |  |  |  |  |  |
| *Groups* | *Count* | *Sum* | *Average* | *Variance* |  |  |
| Brazil | 6 | 4155.465 | 692.577 | 3188.568 |  |  |
| Ethiopian Ardi | 6 | 5050.528 | 841.755 | 789.902 |  |  |
| Ethiopian Yirgz | 6 | 3807.529 | 634.588 | 15923.588 |  |  |
| Myanmar | 6 | 3569.851 | 594.975 | 2268.733 |  |  |
| Columbia | 6 | 4063.410 | 677.235 | 730.034 |  |  |
| Mexico | 6 | 4326.052 | 721.009 | 2593.064 |  |  |
| **ANOVA** |  |  |  |  |  |  |
| *Source of Variation* | *SS* | *df* | *MS* | *F* | *P-value* | *F crit* |
| Between Groups | 217074.60 | 5 | 43414.92 | 10.22 | 8.66E-06 | 2.53355 |
| Within Groups | 127469.45 | 30 | 4248.98 |  |  |  |
|  |  |  |  |  |  |  |
| Total | 344544.05 | 35 |  |  |  |  |

**Table S7a**. One way ANOVA for the comparison of the 3-CQA concentration of the six coffee samples using the cold brew method.

| ***Analysis of Variance (One-Way)*** | | |  |  |  |  |
| --- | --- | --- | --- | --- | --- | --- |
|  |  |  |  |  |  |  |
| **SUMMARY** |  |  |  |  |  |  |
| *Groups* | *Count* | *Sum* | *Average* | *Variance* |  |  |
| Brazil | 8 | 4103.796 | 512.974 | 784.531 |  |  |
| Ethiopian Ardi | 8 | 3712.037 | 464.005 | 188.594 |  |  |
| Ethiopian Yirgz | 8 | 3071.487 | 383.936 | 2361.188 |  |  |
| Myanmar | 8 | 2843.555 | 355.444 | 794.825 |  |  |
| Columbia | 8 | 3251.085 | 406.386 | 3562.110 |  |  |
| Mexico | 8 | 2750.449 | 343.806 | 2528.099 |  |  |
| **ANOVA** |  |  |  |  |  |  |
| *Source of Variation* | *SS* | *df* | *MS* | *F* | *P-value* | *F crit* |
| Between Groups | 172507.551 | 5 | 34501.510 | 20.257 | 3.10E-10 | 2.43769 |
| Within Groups | 71535.4288 | 42 | 1703.224 |  |  |  |
|  |  |  |  |  |  |  |
| Total | 244042.98 | 47 |  |  |  |  |

**Table S7b**. One way ANOVA for the comparison of the 3-CQA concentration of the six coffee samples using the hot brew method.

| ***Analysis of Variance (One-Way)*** | | |  |  |  |  |
| --- | --- | --- | --- | --- | --- | --- |
|  |  |  |  |  |  |  |
| **SUMMARY** |  |  |  |  |  |  |
| *Groups* | *Count* | *Sum* | *Average* | *Variance* |  |  |
| Brazil | 6 | 3299.928 | 549.988 | 1105.307 |  |  |
| Ethiopian Ardi | 6 | 4244.727 | 707.454 | 1855.544 |  |  |
| Ethiopian Yirgz | 6 | 3061.820 | 510.303 | 9423.842 |  |  |
| Myanmar | 6 | 2935.135 | 489.189 | 1432.162 |  |  |
| Columbia | 6 | 3373.701 | 562.283 | 1167.786 |  |  |
| Mexico | 6 | 3666.756 | 611.126 | 2277.959 |  |  |
| **ANOVA** |  |  |  |  |  |  |
| *Source of Variation* | *SS* | *df* | *MS* | *F* | *P-value* | *F crit* |
| Between Groups | 186728.168 | 5 | 37345.63 | 12.98 | 9.15E-07 | 2.5336 |
| Within Groups | 86312.9991 | 30 | 2877.10 |  |  |  |
|  |  |  |  |  |  |  |
| Total | 273041.167 | 35 |  |  |  |  |

**Table S8a**. One way ANOVA for the comparison of the total CQA concentration of the six coffee samples using the cold brew method.

| ***Analysis of Variance (One-Way)*** | | |  |  |  |  |
| --- | --- | --- | --- | --- | --- | --- |
|  |  |  |  |  |  |  |
| **SUMMARY** |  |  |  |  |  |  |
| *Groups* | *Count* | *Sum* | *Average* | *Variance* |  |  |
| Brazil | 8 | 4510.162 | 563.770 | 1114.946 |  |  |
| Ethiopian Ardi | 8 | 4412.906 | 551.613 | 418.631 |  |  |
| Ethiopian Yirgz | 8 | 3843.415 | 480.427 | 4324.674 |  |  |
| Myanmar | 8 | 3430.989 | 428.874 | 1672.066 |  |  |
| Columbia | 8 | 3901.144 | 487.643 | 5442.880 |  |  |
| Mexico | 8 | 3324.372 | 415.546 | 3962.860 |  |  |
| **ANOVA** |  |  |  |  |  |  |
| *Source of Variation* | *SS* | *df* | *MS* | *F* | *P-value* | *F crit* |
| Between Groups | 2191465.8 | 5 | 438293.15 | 10.09 | 2.14E-06 | 2.4377 |
| Within Groups | 1823709.0 | 42 | 43421.64 |  |  |  |
|  |  |  |  |  |  |  |
| Total | 4015174.8 | 47 |  |  |  |  |

**Table S8b**. One way ANOVA for the comparison of the total CQA concentration of the six coffee samples using the hot brew method.

| ***Analysis of Variance (One-Way)*** | | |  |  |  |  |
| --- | --- | --- | --- | --- | --- | --- |
|  |  |  |  |  |  |  |
| **SUMMARY** |  |  |  |  |  |  |
| *Groups* | *Count* | *Sum* | *Average* | *Variance* |  |  |
| Brazil | 6 | 15025.724 | 2504.287 | 35522.304 |  |  |
| Ethiopian Ardi | 6 | 19620.267 | 3270.045 | 27720.477 |  |  |
| Ethiopian Yirgz | 6 | 15161.236 | 2526.873 | 254272.193 |  |  |
| Myanmar | 6 | 14178.054 | 2363.009 | 42074.610 |  |  |
| Columbia | 6 | 16015.603 | 2669.267 | 14487.442 |  |  |
| Mexico | 6 | 16854.546 | 2809.091 | 42567.755 |  |  |
| **ANOVA** |  |  |  |  |  |  |
| *Source of Variation* | *SS* | *df* | *MS* | *F* | *P-value* | *F crit* |
| Between Groups | 3114515.44 | 5 | 622903.09 | 8.97 | 2.69E-05 | 2.5336 |
| Within Groups | 2083223.9 | 30 | 69440.80 |  |  |  |
|  |  |  |  |  |  |  |
| Total | 5197739.34 | 35 |  |  |  |  |

**Table S6**. Two-tailed student’s t-tests for the comparison of the pH values between cold and hot brewing methods.

| **Brazil** |  |  |  | **Ethiopian Ardi** |  |  |
| --- | --- | --- | --- | --- | --- | --- |
|  | *Cold* | *Hot* |  |  | *Cold* | *Hot* |
| Mean | 5.035 | 5.1016667 |  | Mean | 4.9633333 | 4.9183333 |
| Variance | 0.03899 | 0.0002567 |  | Variance | 0.0102267 | 0.0009367 |
| Observations | 6 | 6 |  | Observations | 6 | 6 |
| Pooled Variance | 0.0196233 |  |  | Pooled Variance | 0.0055817 |  |
| Hypothesized Mean Difference | 0 |  |  | Hypothesized Mean Difference | 0 |  |
| df | 10 |  |  | df | 10 |  |
| t Stat | -0.8242956 |  |  | t Stat | 1.0432567 |  |
| P(T<=t) two-tail | 0.4289942 |  |  | P(T<=t) two-tail | 0.3213935 |  |
| t Critical two-tail | 2.2281389 |  |  | t Critical two-tail | 2.2281389 |  |
|  |  |  |  |  |  |  |
| **Columbia** |  |  |  | **Mexico** |  |  |
|  | *Cold* | *Hot* |  |  | *Cold* | *Hot* |
| Mean | 4.9966667 | 4.8483333 |  | Mean | 5.075 | 4.9916667 |
| Variance | 0.0040267 | 0.0153767 |  | Variance | 0.00215 | 0.0020967 |
| Observations | 6 | 6 |  | Observations | 6 | 6 |
| Pooled Variance | 0.0097017 |  |  | Pooled Variance | 0.0021233 |  |
| Hypothesized Mean Difference | 0 |  |  | Hypothesized Mean Difference | 0 |  |
| df | 10 |  |  | df | 10 |  |
| t Stat | 2.6084121 |  |  | t Stat | 3.1323501 |  |
| P(T<=t) two-tail | 0.0261118 |  |  | P(T<=t) two-tail | 0.0106478 |  |
| t Critical two-tail | 2.2281389 |  |  | t Critical two-tail | 2.2281389 |  |
|  |  |  |  |  |  |  |
| **Ethiopian Yirgz** |  |  |  | **Myanmar** |  |  |
|  | *Cold* | *Hot* |  |  | *Cold* | *Hot* |
| Mean | 5.0116667 | 5.01 |  | Mean | 5.1316667 | 4.9466667 |
| Variance | 0.0007767 | 0.05356 |  | Variance | 0.0019767 | 0.0012667 |
| Observations | 6 | 6 |  | Observations | 6 | 6 |
| Pooled Variance | 0.0271683 |  |  | Pooled Variance | 0.0016217 |  |
| Hypothesized Mean Difference | 0 |  |  | Hypothesized Mean Difference | 0 |  |
| df | 10 |  |  | df | 10 |  |
| t Stat | 0.0175137 |  |  | t Stat | 7.9570404 |  |
| P(T<=t) two-tail | 0.9863713 |  |  | P(T<=t) two-tail | 1.2343E-05 |  |
| t Critical two-tail | 2.2281389 |  |  | t Critical two-tail | 2.2281389 |  |

**Table S7**. Two-tailed student’s t-tests for the comparison of the total acidity titrated to pH of 6.0 between cold and hot brewing methods.

| **Brazil** |  |  |  | **Ethiopian Ardi** |  |  |
| --- | --- | --- | --- | --- | --- | --- |
|  | *Cold* | *Hot* |  |  | *Cold* | *Hot* |
| Mean | 2.833333 | 3.166667 |  | Mean | 2.583333 | 3.833333 |
| Variance | 0.066667 | 0.062667 |  | Variance | 0.049667 | 0.166667 |
| Observations | 6 | 6 |  | Observations | 6 | 6 |
| Pooled Variance | 0.064667 |  |  | Pooled Variance | 0.108167 |  |
| Hypothesized Mean Difference | 0 |  |  | Hypothesized Mean Difference | 0 |  |
| df | 10 |  |  | df | 10 |  |
| t Stat | -2.27038 |  |  | t Stat | -6.58300 |  |
| P(T<=t) two-tail | 0.04654 |  |  | P(T<=t) two-tail | 6.21E-05 |  |
| t Critical two-tail | 2.22814 |  |  | t Critical two-tail | 2.228139 |  |
|  |  |  |  |  |  |  |
| **Columbia** |  |  |  | **Mexico** |  |  |
|  | *Cold* | *Hot* |  |  | *Cold* | *Hot* |
| Mean | 2.933333 | 4.266667 |  | Mean | 2.133333 | 3.583333 |
| Variance | 0.054667 | 0.066667 |  | Variance | 0.018667 | 0.257667 |
| Observations | 6 | 6 |  | Observations | 6 | 6 |
| Pooled Variance | 0.0606667 |  |  | Pooled Variance | 0.1381667 |  |
| Hypothesized Mean Difference | 0 |  |  | Hypothesized Mean Difference | 0 |  |
| df | 10 |  |  | df | 10 |  |
| t Stat | -9.37614 |  |  | t Stat | -6.75658 |  |
| P(T<=t) two-tail | 2.86E-06 |  |  | P(T<=t) two-tail | 5.00E-05 |  |
| t Critical two-tail | 2.228139 |  |  | t Critical two-tail | 2.228139 |  |
|  |  |  |  |  |  |  |
| **Ethiopian Yirgz** |  |  |  | **Myanmar** |  |  |
|  | *Cold* | *Hot* |  |  | *Cold* | *Hot* |
| Mean | 2.55 | 3.616667 |  | Mean | 2.516667 | 3.183333 |
| Variance | 0.047 | 0.153667 |  | Variance | 0.029667 | 0.877667 |
| Observations | 6 | 6 |  | Observations | 6 | 6 |
| Pooled Variance | 0.100333 |  |  | Pooled Variance | 0.453667 |  |
| Hypothesized Mean Difference | 0 |  |  | Hypothesized Mean Difference | 0 |  |
| df | 10 |  |  | df | 10 |  |
| t Stat | -5.83266 |  |  | t Stat | -1.71436 |  |
| P(T<=t) two-tail | 0.00017 |  |  | P(T<=t) two-tail | 0.11723 |  |
| t Critical two-tail | 2.22814 |  |  | t Critical two-tail | 2.22814 |  |

**Table S8**. Two-tailed student’s t-tests for the comparison of the total acidity titrated to pH of 8.0 between cold and hot brewing methods.

| **Brazil** |  |  |  | **Ethiopian Ardi** |  |  |
| --- | --- | --- | --- | --- | --- | --- |
|  | *Cold* | *Hot* |  |  | *Cold* | *Hot* |
| Mean | 5.88333 | 6.53333 |  | Mean | 5.18333 | 7.45 |
| Variance | 0.14967 | 0.21867 |  | Variance | 0.02967 | 0.551 |
| Observations | 6 | 6 |  | Observations | 6 | 6 |
| Pooled Variance | 0.18417 |  |  | Pooled Variance | 0.29033 |  |
| Hypothesized Mean Difference | 0 |  |  | Hypothesized Mean Difference | 0 |  |
| df | 10 |  |  | df | 10 |  |
| t Stat | -2.62342 |  |  | t Stat | -7.28618 |  |
| P(T<=t) two-tail | 0.02545 |  |  | P(T<=t) two-tail | 2.64E-05 |  |
| t Critical two-tail | 2.22814 |  |  | t Critical two-tail | 2.228139 |  |
|  |  |  |  |  |  |  |
| **Columbia** |  |  |  | **Mexico** |  |  |
|  | *Cold* | *Hot* |  |  | *Cold* | *Hot* |
| Mean | 5.516667 | 7.85 |  | Mean | 4.75 | 6.683333 |
| Variance | 0.161667 | 0.007 |  | Variance | 0.115 | 0.601667 |
| Observations | 6 | 6 |  | Observations | 6 | 6 |
| Pooled Variance | 0.084333 |  |  | Pooled Variance | 0.358333 |  |
| Hypothesized Mean Difference | 0 |  |  | Hypothesized Mean Difference | 0 |  |
| df | 10 |  |  | df | 10 |  |
| t Stat | -13.9167 |  |  | t Stat | -5.59402 |  |
| P(T<=t) two-tail | 7.17E-08 |  |  | P(T<=t) two-tail | 0.000230 |  |
| t Critical two-tail | 2.228139 |  |  | t Critical two-tail | 2.228139 |  |
|  |  |  |  |  |  |  |
| **Ethiopian Yirgz** |  |  |  | **Myanmar** |  |  |
|  | *Cold* | *Hot* |  |  | *Cold* | *Hot* |
| Mean | 5.25 | 7.083333 |  | Mean | 5.316667 | 6.4 |
| Variance | 0.059 | 0.837667 |  | Variance | 0.069667 | 0.98 |
| Observations | 6 | 6 |  | Observations | 6 | 6 |
| Pooled Variance | 0.448333 |  |  | Pooled Variance | 0.524833 |  |
| Hypothesized Mean Difference | 0 |  |  | Hypothesized Mean Difference | 0 |  |
| df | 10 |  |  | df | 10 |  |
| t Stat | -4.74244 |  |  | t Stat | -2.59007 |  |
| P(T<=t) two-tail | 0.00079 |  |  | P(T<=t) two-tail | 0.02695 |  |
| t Critical two-tail | 2.22814 |  |  | t Critical two-tail | 2.22814 |  |

**Table S9**. Two-tailed student’s t-tests for the comparison of the antioxidant activity between cold and hot brewing methods.

| **Brazil** |  |  |  | **Ethiopian Ardi** |  |  |
| --- | --- | --- | --- | --- | --- | --- |
|  | *Cold* | *Hot* |  |  | *Cold* | *Hot* |
| Mean | 18.339416 | 16.109489 |  | Mean | 19.948905 | 17.445255 |
| Variance | 5.464686 | 9.142544 |  | Variance | 2.618339 | 4.203357 |
| Observations | 6 | 6 |  | Observations | 6 | 6 |
| Pooled Variance | 7.3036150 |  |  | Pooled Variance | 3.4108477 |  |
| Hypothesized Mean Difference | 0 |  |  | Hypothesized Mean Difference | 0 |  |
| df | 10 |  |  | df | 10 |  |
| P(T<=t) two-tail | 0.1834425 |  |  | P(T<=t) two-tail | 0.0407776 |  |
| t Critical two-tail | 2.2281389 |  |  | t Critical two-tail | 2.2281389 |  |
|  |  |  |  |  |  |  |
| **Columbia** |  |  |  | **Mexico** |  |  |
|  | *Cold* | *Hot* |  |  | *Variable 1* | *Variable 2* |
| Mean | 19.983316 | 15.328467 |  | Mean | 20.182482 | 14.690302 |
| Variance | 6.266747 | 3.693409 |  | Variance | 2.730098 | 10.175837 |
| Observations | 7 | 6 |  | Observations | 6 | 7 |
| Pooled Variance | 5.0970480 |  |  | Pooled Variance | 6.7914100 |  |
| Hypothesized Mean Difference | 0 |  |  | Hypothesized Mean Difference | 0 |  |
| df | 11 |  |  | df | 11 |  |
| P(T<=t) two-tail | 0.0034655 |  |  | P(T<=t) two-tail | 0.0030048 |  |
| t Critical two-tail | 2.2009852 |  |  | t Critical two-tail | 2.2009852 |  |
|  |  |  |  |  |  |  |
| **Ethiopian Yirgz** |  |  |  | **Myanmar** |  |  |
|  | *Cold* | *Hot* |  |  | *Cold* | *Hot* |
| Mean | 20.715328 | 13.364964 |  | Mean | 19.720542 | 13.364964 |
| Variance | 9.756769 | 0.974692 |  | Variance | 1.426212 | 8.113698 |
| Observations | 6 | 6 |  | Observations | 7 | 6 |
| Pooled Variance | 5.3657307 |  |  | Pooled Variance | 4.4659784 |  |
| Hypothesized Mean Difference | 0 |  |  | Hypothesized Mean Difference | 0 |  |
| df | 10 |  |  | df | 11 |  |
| P(T<=t) two-tail | 0.0002632 |  |  | P(T<=t) two-tail | 0.0002148 |  |
| t Critical two-tail | 2.2281389 |  |  | t Critical two-tail | 2.2009852 |  |

**Table S10**. Two-tailed student’s t-tests for the comparison of the 5-CQA concentration between cold and hot brewing methods.

| **Brazil** |  |  |  | **Ethiopian Ardi** |  |  |
| --- | --- | --- | --- | --- | --- | --- |
|  | *Cold* | *Hot* |  |  | *Cold* | *Hot* |
| Mean | 1124.16 | 1260.92 |  | Mean | 1133.46 | 1720.60 |
| Variance | 3907.84 | 12235.09 |  | Variance | 1320.35 | 9928.72 |
| Observations | 8 | 6 |  | Observations | 8 | 6 |
| Pooled Variance | 7377.53 |  |  | Pooled Variance | 4907.17 |  |
| Hypothesized Mean Difference | 0 |  |  | Hypothesized Mean Difference | 0 |  |
| df | 12 |  |  | df | 12 |  |
| t Stat | -2.9483 |  |  | t Stat | -15.520 |  |
| P(T<=t) two-tail | 0.0122 |  |  | P(T<=t) two-tail | 2.63E-09 |  |
| t Critical two-tail | 2.1788 |  |  | t Critical two-tail | 2.17881 |  |
|  |  |  |  |  |  |  |
| **Columbia** |  |  |  | **Mexico** |  |  |
|  | *Cold* | *Hot* |  |  | *Cold* | *Hot* |
| Mean | 1017.64 | 1429.43 |  | Mean | 856.64 | 1475.68 |
| Variance | 24546.23 | 4431.71 |  | Variance | 19065.71 | 12425.34 |
| Observations | 8 | 6 |  | Observations | 8 | 6 |
| Pooled Variance | 16165.2 |  |  | Pooled Variance | 16298.9 |  |
| Hypothesized Mean Difference | 0 |  |  | Hypothesized Mean Difference | 0 |  |
| df | 12 |  |  | df | 12 |  |
| t Stat | -5.9971 |  |  | t Stat | -8.9783 |  |
| P(T<=t) two-tail | 6.24E-05 |  |  | P(T<=t) two-tail | 1.13E-06 |  |
| t Critical two-tail | 2.17881 |  |  | t Critical two-tail | 2.17881 |  |
|  |  |  |  |  |  |  |
| **Ethiopian Yirgz** |  |  |  | **Myanmar** |  |  |
|  | *Cold* | *Hot* |  |  | *Cold* | *Hot* |
| Mean | 1030.54 | 1385.37 |  | Mean | 912.21 | 1433.19 |
| Variance | 15916.91 | 81187.67 |  | Variance | 15956.78 | 116067.38 |
| Observations | 8 | 6 |  | Observations | 8 | 6 |
| Pooled Variance | 43113.1 |  |  | Pooled Variance | 57669.5 |  |
| Hypothesized Mean Difference | 0 |  |  | Hypothesized Mean Difference | 0 |  |
| df | 12 |  |  | df | 12 |  |
| t Stat | -3.16425 |  |  | t Stat | -4.01705 |  |
| P(T<=t) two-tail | 0.00816 |  |  | P(T<=t) two-tail | 0.00171 |  |
| t Critical two-tail | 2.17881 |  |  | t Critical two-tail | 2.17881 |  |

**Table S11**. Two-tailed student’s t-tests for the comparison of the 4-CQA concentration between cold and hot brewing methods.

| **Brazil** |  |  |  | **Ethiopian Ardi** |  |  |
| --- | --- | --- | --- | --- | --- | --- |
|  | *Cold* | *Hot* |  |  | *Cold* | *Hot* |
| Mean | 563.770 | 692.577 |  | Mean | 551.613 | 841.755 |
| Variance | 1114.946 | 3188.568 |  | Variance | 418.631 | 789.902 |
| Observations | 8 | 6 |  | Observations | 8 | 6 |
| Pooled Variance | 1978.955 |  |  | Pooled Variance | 573.327 |  |
| Hypothesized Mean Difference | 0 |  |  | Hypothesized Mean Difference | 0 |  |
| df | 12 |  |  | df | 12 |  |
| t Stat | -5.3614 |  |  | t Stat | -22.4370 |  |
| P(T<=t) two-tail | 0.000170 |  |  | P(T<=t) two-tail | 3.63E-11 |  |
| t Critical two-tail | 2.17881 |  |  | t Critical two-tail | 2.17881 |  |
|  |  |  |  |  |  |  |
| **Columbia** |  |  |  | **Mexico** |  |  |
|  | *Cold* | *Hot* |  |  | *Cold* | *Hot* |
| Mean | 487.643 | 677.235 |  | Mean | 415.546 | 721.009 |
| Variance | 5442.880 | 730.034 |  | Variance | 3962.860 | 2593.064 |
| Observations | 8 | 6 |  | Observations | 8 | 6 |
| Pooled Variance | 3479.194 |  |  | Pooled Variance | 3392.112 |  |
| Hypothesized Mean Difference | 0 |  |  | Hypothesized Mean Difference | 0 |  |
| df | 12 |  |  | df | 12 |  |
| t Stat | -5.9516 |  |  | t Stat | -9.7113 |  |
| P(T<=t) two-tail | 6.70E-05 |  |  | P(T<=t) two-tail | 4.91007E-07 |  |
| t Critical two-tail | 2.17881 |  |  | t Critical two-tail | 2.17881 |  |
|  |  |  |  |  |  |  |
| **Ethiopian Yirgz** |  |  |  | **Myanmar** |  |  |
|  | *Cold* | *Hot* |  |  | *Cold* | *Hot* |
| Mean | 480.427 | 634.588 |  | Mean | 428.874 | 594.975 |
| Variance | 4324.674 | 15923.588 |  | Variance | 1672.066 | 2268.733 |
| Observations | 8 | 6 |  | Observations | 8 | 6 |
| Pooled Variance | 9157.555 |  |  | Pooled Variance | 1920.677 |  |
| Hypothesized Mean Difference | 0 |  |  | Hypothesized Mean Difference | 0 |  |
| df | 12 |  |  | df | 12 |  |
| t Stat | -2.9829 |  |  | t Stat | -7.0178 |  |
| P(T<=t) two-tail | 0.0114235 |  |  | P(T<=t) two-tail | 1.40E-05 |  |
| t Critical two-tail | 2.17881 |  |  | t Critical two-tail | 2.17881 |  |

**Table S12**. Two-tailed student’s t-tests for the comparison of the 3-CQA concentration between cold and hot brewing methods.

| **Brazil** |  |  |  | **Ethiopian Ardi** |  |  |
| --- | --- | --- | --- | --- | --- | --- |
|  | *Cold* | *Hot* |  |  | *Cold* | *Hot* |
| Mean | 512.974 | 549.988 |  | Mean | 464.005 | 707.454 |
| Variance | 784.531 | 1105.307 |  | Variance | 188.594 | 1855.544 |
| Observations | 8 | 6 |  | Observations | 8 | 6 |
| Pooled Variance | 918.188 |  |  | Pooled Variance | 883.156 |  |
| Hypothesized Mean Difference | 0 |  |  | Hypothesized Mean Difference | 0 |  |
| df | 12 |  |  | df | 12 |  |
| t Stat | -2.26179 |  |  | t Stat | -15.16866 |  |
| P(T<=t) two-tail | 0.04307 |  |  | P(T<=t) two-tail | 3.42E-09 |  |
| t Critical two-tail | 2.178813 |  |  | t Critical two-tail | 2.178813 |  |
|  |  |  |  |  |  |  |
| **Columbia** |  |  |  | **Mexico** |  |  |
|  | *Cold* | *Hot* |  |  | *Cold* | *Hot* |
| Mean | 406.386 | 562.283 |  | Mean | 343.806 | 611.126 |
| Variance | 3562.110 | 1167.786 |  | Variance | 2528.099 | 2277.959 |
| Observations | 8 | 6 |  | Observations | 8 | 6 |
| Pooled Variance | 2564.475 |  |  | Pooled Variance | 2423.874 |  |
| Hypothesized Mean Difference | 0 |  |  | Hypothesized Mean Difference | 0 |  |
| df | 12 |  |  | df | 12 |  |
| t Stat | -5.70029 |  |  | t Stat | -10.05386 |  |
| P(T<=t) two-tail | 9.909E-05 |  |  | P(T<=t) two-tail | 3.379E-07 |  |
| t Critical two-tail | 2.178813 |  |  | t Critical two-tail | 2.178813 |  |
|  |  |  |  |  |  |  |
| **Ethiopian Yirgz** |  |  |  | **Myanmar** |  |  |
|  | *Cold* | *Hot* |  |  | *Cold* | *Hot* |
| Mean | 383.936 | 510.303 |  | Mean | 355.444 | 489.189 |
| Variance | 2361.188 | 9423.842 |  | Variance | 794.825 | 1432.162 |
| Observations | 8 | 6 |  | Observations | 8 | 6 |
| Pooled Variance | 5303.961 |  |  | Pooled Variance | 1060.382 |  |
| Hypothesized Mean Difference | 0 |  |  | Hypothesized Mean Difference | 0 |  |
| df | 12 |  |  | df | 12 |  |
| t Stat | -3.21286 |  |  | t Stat | -7.60505 |  |
| P(T<=t) two-tail | 0.007453 |  |  | P(T<=t) two-tail | 6.289E-06 |  |
| t Critical two-tail | 2.178813 |  |  | t Critical two-tail | 2.178813 |  |

**Table S13**. Two-tailed student’s t-tests for the comparison of the total CQA concentration between cold and hot brewing methods.

| **Brazil** |  |  |  | **Ethiopian Ardi** |  |  |
| --- | --- | --- | --- | --- | --- | --- |
|  | *Cold* | *Hot* |  |  | *Cold* | *Hot* |
| Mean | 2200.965 | 2504.287 |  | Mean | 2149.079 | 3270.045 |
| Variance | 13119.184 | 35522.304 |  | Variance | 4904.527 | 27720.477 |
| Observations | 8 | 6 |  | Observations | 8 | 6 |
| Pooled Variance | 22453.82 |  |  | Pooled Variance | 14411.17 |  |
| Hypothesized Mean Difference | 0 |  |  | Hypothesized Mean Difference | 0 |  |
| df | 12 |  |  | df | 12 |  |
| t Stat | -3.74815 |  |  | t Stat | -17.2902 |  |
| P(T<=t) two-tail | 0.00278 |  |  | P(T<=t) two-tail | 7.58E-10 |  |
| t Critical two-tail | 2.17881 |  |  | t Critical two-tail | 2.178813 |  |
|  |  |  |  |  |  |  |
| **Columbia** |  |  |  | **Mexico** |  |  |
|  | *Cold* | *Hot* |  |  | *Cold* | *Hot* |
| Mean | 1911.732 | 2669.267 |  | Mean | 1616.050 | 2809.091 |
| Variance | 83976.825 | 14487.442 |  | Variance | 63047.299 | 42567.755 |
| Observations | 8 | 6 |  | Observations | 8 | 6 |
| Pooled Variance | 55022.92 |  |  | Pooled Variance | 54514.16 |  |
| Hypothesized Mean Difference | 0 |  |  | Hypothesized Mean Difference | 0 |  |
| df | 12 |  |  | df | 12 |  |
| t Stat | -5.9798172 |  |  | t Stat | -9.4614450 |  |
| P(T<=t) two-tail | 6.412E-05 |  |  | P(T<=t) two-tail | 6.492E-07 |  |
| t Critical two-tail | 2.1788128 |  |  | t Critical two-tail | 2.17881283 |  |
|  |  |  |  |  |  |  |
| **Ethiopian Yirgz** |  |  |  | **Myanmar** |  |  |
|  | *Cold* | *Hot* |  |  | *Cold* | *Hot* |
| Mean | 1895.028 | 2526.873 |  | Mean | 1696.704 | 2363.009 |
| Variance | 57621.637 | 254272.193 |  | Variance | 37860.385 | 42074.610 |
| Observations | 8 | 6 |  | Observations | 8 | 6 |
| Pooled Variance | 139559.37 |  |  | Pooled Variance | 39616.31 |  |
| Hypothesized Mean Difference | 0 |  |  | Hypothesized Mean Difference | 0 |  |
| df | 12 |  |  | df | 12 |  |
| t Stat | -3.1317543 |  |  | t Stat | -6.1985861 |  |
| P(T<=t) two-tail | 0.0086636 |  |  | P(T<=t) two-tail | 4.596E-05 |  |
| t Critical two-tail | 2.1788128 |  |  | t Critical two-tail | 2.1788128 |  |
